# Assessment of the Chemical Diversity and Functional Properties of Secondary Metabolites from the Marine Fungus *Asteromyces cruciatus*

**DOI:** 10.3390/jof11010003

**Published:** 2024-12-24

**Authors:** María Paz González-Troncoso, Catalina Landeta-Salgado, Javiera Munizaga, Ruth Hornedo-Ortega, María del Carmen García-Parrilla, María Elena Lienqueo

**Affiliations:** 1 Department of Chemical Engineering, Biotechnology, and Materials, Centre for Biotechnology and Bioengineering (CeBiB), University of Chile, Beauchef 851, Santiago 8370456, Chile; maria.gonzalez.t@ug.uchile.cl (M.P.G.-T.); cmlandeta@uc.cl (C.L.-S.); jmmunizaga@uc.cl (J.M.); 2 Departamento de Nutrición y Bromatología, Toxicología y Medicina Legal, Facultad de Farmacia, Universidad de Sevilla, C/Profesor García González nº 2, 41012 Sevilla, Spain; rhornedo@us.es (R.H.-O.); mcparrilla@us.es (M.d.C.G.-P.)

**Keywords:** anthraquinone compounds, bioactive compounds, phenolic compounds, metabolome, marine-derived fungi, *Asteromyces cruciatus*

## Abstract

Natural compounds derived from microorganisms, especially those with antioxidant and anticancer properties, are gaining attention for their potential applications in biomedical, cosmetic, and food industries. Marine fungi, such as *Asteromyces cruciatus*, are particularly promising due to their ability to produce bioactive metabolites through the degradation of marine algal polysaccharides. This study investigates the metabolic diversity of *A. cruciatus* grown on different carbon sources: glucose, *Durvillaea* spp., and *Macrocystis pyrifera*. Crude extracts of fungal biomass were analyzed for total phenolic content (TPC), antioxidant capacity (TAC), toxicity, and phenolic compound identification using ultra-high-performance liquid chromatography coupled with high-resolution electrospray ionization mass spectrometry (UHPLC-MS/MS). The analysis revealed the presence of anthraquinone compounds, including emodin (0.36 ± 0.08 mg/g DW biomass) and citrereosein in glucose medium and citrereosein and endocrocin in *M. pyrifera* medium. No such compounds were detected in *Durvillaea* spp. medium. The glucose-grown extract exhibited the highest TPC (3.09 ± 0.04 mg GAE/g DW) and TAC (39.70 ± 1.0 µmol TEq/g biomass). Additionally, no detrimental effects were observed on a neuronal cell line. These findings highlight the influence of carbon sources on the production of bioactive metabolites and their functional properties, providing valuable insights into the biotechnological potential of *A. cruciatus*.

## 1. Introduction

Natural compounds with health-promoting and non-toxic properties, especially those derived from microorganisms, have gained significant attention in recent years. Microbial production of bio-compounds is advantageous due to the chemical diversity of metabolites, such as alkaloids, terpenoids, lipids, enzymes, and polyketides [1,2,3], which can be synthesized via optimized fermentation processes using unconventional, low-cost raw materials [4,5,6].

Marine-derived fungi refer to fungi isolated from marine or marine-related habitats, including marine animals, sea foam, seaweed, and mangroves, and they represent a largely underexplored source of bioactive compounds [7,8]. These fungi degrade seaweed, particularly brown algae, to access polysaccharides, such as laminarin, fucoidan, cellulose, and alginate, which serve as raw materials to produce bioactive metabolites with antioxidant, antimicrobial, anti-inflammatory, neuroprotective, and anticancer effects [9,10,11]. Some methods have been developed for quantifying fungal growth. These include the measurement of the hyphal filament length, the detection of chitin, a cell wall component, and the quantification of ergosterol, a major membrane component. The latter method is considered the preferred approach due to its higher sensitivity, as it is a specific indicator of fungal presence and is less labor-intensive [12,13]. Marine-derived fungi also exhibit notable sensitivity to abiotic factors, including variations in nutrient availability, salinity, temperature, osmotic stress, etc. [14,15,16], providing opportunities for the discovery of novel compounds with potential industrial applications.

The vast Chilean coast is host to several species of macroalgae, such as the brown macroalgae *Durvillaes* spp. and *Macrocysits* spp. These renewable natural resources have been little exploited and explored, being mainly used for direct consumption or as a source of alginate [17,18]. The biorefinery of brown algae, particularly in microbial compound production, primarily involves the degradation of carbohydrates in the algal cell wall. Polysaccharides, such as laminarin, fucoidan, cellulose, and alginate, can account for over 50% of the total dry weight of brown algae, with proteins (4–24%) and lipids (approximately 4%) being present in smaller quantities [19]. The degradation of these polysaccharides by extracellular enzymes, including fucoidanases, cellulases, and alginate lyases, releases [20] monosaccharides, which impact the growth of microorganisms and facilitate the production of bioactive compounds [4,5].

On the other hand, *Asteromyces cruciatus* is an underexplored marine-derived fungus isolated from various marine sources, such as macroalgae, sponges, and sea foam. It has been reported that this fungus can enzymatically degrade alginate [21], a polysaccharide found in the cell walls of brown seaweeds like *Durvillaea* spp. and *Macrocystis* spp. Chemical studies have shown that *A. cruciatus,* under conditions like variation in media composition, cultivation periods, osmotic stress, UV-light exposure, and temperature, synthesizes a range of metabolites. These metabolites include gliovictin [22], Lajollamide A [23], primarolides A and B [14], and the six polyketides acrucipentyns A–F and anthraquinone compounds, which exhibit antimicrobial activity against *Staphylococcus aureus* [24,25].

**Figure 1:**
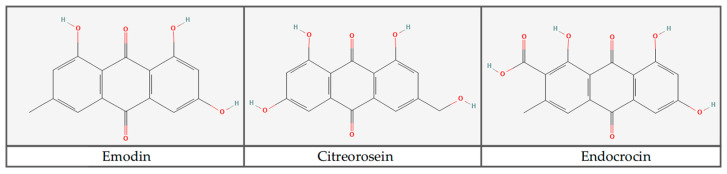
Chemical structure depiction of the anthraquinone compounds emodin, citreorosein, and endocrocin from the PubChem Compound Summary [29,30,31].

Anthraquinones, a group of polyketides, are structurally diverse compounds produced by plants, insects, and micro-organisms known for their stability to light, temperature, and oxidation, as well as their antibiotic, antioxidant, and anticancer properties [26,27]. These compounds have a wide range of industrial applications, particularly as natural dyes and in the pharmaceutical industry [28]. Some examples of anthraquinones and their chemical structures, which consist of two aromatic rings joined by two carbonyl groups to form a planar aromatic structure, are shown in Figure 1.

This study investigates the metabolic diversity of *A. cruciatus* cultured on three different carbon sources: glucose, *Durvillaea* spp., and *Macrocystis pyrifera*. This work aims to identify bioactive compounds produced under these conditions and assess how carbon sources influence the fungal metabolome. We identified differences in the fungal metabolome, including the presence of anthraquinone compounds, in a specific culture medium.

## 2. Materials and Methods

### 2.1. Fungal and Seaweed Strain

The marine fungus *Asteromyces cruciatus* 32142, isolated from sea foam in Souya, Hokkaido, Japan, is part of the fungal collection at the National Institute of Biological Resources and Technology Assessment and Technology Center (NBRC (http://www.nite.go.jp/en/nbrc/, accessed in 15 March 2021)), Japan.

Pulverized *Durvillaea* spp. was donated by Herbamar™ (Concepción, Chile). *Macrocystis pyrifera* was collected in Puerto Montt, Los Lagos Region, Chile and kindly provided by Dr. Buschmann (Universidad de Los Lagos). The seaweeds were harvested in October 2023, dried at 40 °C, ground, and sieved to a particle size of 0.22 mm.

### 2.2. Cultivation of Fungus

*A. cruciatus* was maintained on a modified potato dextrose agar (PDA) medium containing 1% sodium alginate and 1% sodium chloride.

For liquid cultures, fungal conditions were maintained for 2, 3, 4, 5, and 6 days in three different culture media: marine–glucose medium or seaweed-based media with *Durvillaea* spp. or *M. pyrifera*. The marine–glucose medium consisted of 20 g/L of glucose, 6 g/L of yeast extract, and artificial seawater, and the seaweed medium was based on Landeta et al., 2021 [32] with slight modifications. The medium was prepared using 30 g/L of *Durvillaea* spp. or *M. pyrifera*, 6 g/L of yeast extract and additional minerals. The pH of the culture medium was adjusted to 5.0 using HCl or NaOH.

Fungal inoculums were prepared by growing fungus in a conical flask (125 mL) containing 50 mL of medium and inoculated with 5 pieces (0.66 cm^2^) of 10-day-old PDA-modified culture. The cultures were incubated at 22 °C and 150 rpm in the dark. Mycelial biomass was collected through filtration using a Miracloth filter (Merck, Darmstadt, Germany), washed twice with distilled water, lyophilized, ground with a mortar, and stored at −20 °C until further analysis. The culture was performed in biological triplicates.

For clarity, the following abbreviations will be used throughout the manuscript. *A. cruciatus* grown on glucose, *Durvillaea* spp., and *M. pyrifera* media will be referred to as AG, AD, and AM, respectively, and the corresponding media will be referred to as Glucose G, *Durvillaea* spp. D, and *M. pyrifera* M.

### 2.3. Ergosterol Analysis of Fungal Biomass

Ergosterol extraction was performed under light protection following the method of Landeta-Salgado et al., 2024 [33] with slight modifications. A 0.1 g sample of dry powdered mycelium was vortexed with hexane (1:60 *w*/*v*) for 1 min, followed by 1 min of incubation on ice. This process was repeated twice. The extract was centrifuged at 6000× *g* for 15 min at 4 °C, and the supernatant was collected. The extraction process was repeated three times, and the combined supernatants were filtered through a 0.22 µm PTFE filter (Merck, Darmstadt, Germany). The extract was concentrated using a vacuum concentrator centrifuge at 30 °C and reconstituted in 500 µL of methanol. The ergosterol content was quantified using high-performance liquid chromatography (HPLC) with a photodiode array detector (DAD) (Shimadzu, Kyoto, Japan) and an Onyx Monolithic C18 reverse phase column (5 µm × 100 mm, 4.6 mm, Phenomenex, Torrance, CA, USA), as described by Souilem et al., 2017 [34]. The ergosterol concentration was determined using an external standard curve (Sigma Aldrich, Cod. PHR1512, St. Louis, MO, USA) with concentrations ranging from 0.05 to 0.3 mg/mL. Extraction was performed in technical duplicate.

### 2.4. Glucose Concentration of the Fungal Culture Medium

The glucose variation in the fungus growing on glucose medium was measured using the sugar reduction method based on the dinitrosalicylic acid (DNS) assay described by Miller 1959 [35], with glucose as the standard. One milliliter of supernatants from cultures grown for 2, 3, 4, 5, and 6 days was filtered through PVDF (Millipore, Sigma Aldrich, USA). A 100 µL aliquot of the supernatant was mixed with 100 µL of DNS reagent and incubated for 5 min at 95 °C in a thermocycler. After cooling on ice for 5 min, 150 µL of the reaction mixture was transferred to a 96-well plate, and absorbance was measured at 550 nm using a microplate reader. The glucose variation was estimated using a glucose standard curve with concentrations of 0.25, 0.5, 0.75, and 1 mg/mL.

### 2.5. Characterization of Crude Extracts

#### 2.5.1. Untargeted Metabolomics Using UHPLC-QTOF-MS/MS

##### Preparation of Crude Extracts

The extraction was carried out following the protocol described by Landeta-Salgado et al., 2024 [33] with modifications. Crude extracts were prepared from 6-day-old fungal biomass (0.3 g) through extraction with 70% methanol (1:20 *w*/*v*). The samples were sonicated using a Q500 Sonicator^®^ (QSonica, Newtown, CT, USA) for 2.5 min, with cycles of 10 s on and 10 s off at 40% amplitude using a 3.2 mm probe. The mixture was then incubated overnight at 4 °C. After centrifugation at 8000× *g* for 10 min at 4 °C, supernatants were collected. The residues were re-extracted once more, and the combined supernatants were filtered through a PTFE filter (Merck, Darmstadt, Germany). The solvent was evaporated using a vacuum concentrator at 30 °C and then freeze-dried. Extraction was performed in technical duplicate.

##### UHPLC-MS/MS Analysis

Analysis was performed on a Kinetex C18 column (100 mm × 2.1 mm, 1.7 μm particle size) at 40 °C, with a sample injection volume of 10 μL and a flow rate of 0.4 mL/min. The mobile phases consisted of (A) 0.1% (*v*/*v*) formic acid in water and (B) 0.1% (*v*/*v*) formic acid, 9.9% water, and 90% (*v*/*v*) acetonitrile. The gradient elution conditions were as follows: 0–1 min, 22% B, 1–10 min, 22–99% B, 10.01–12.5 min, 99% B, 12.51–13.01 min, 99–22% B, and 13.01–16.00 min, 22% B. Ionization was carried out in negative electrospray ionization (ESI) mode with a capillary voltage of 4500 V, a pressure of 2 bars, a dry gas temperature of 250 °C, and flow rate of 8 L/min. Data acquisition and processing were performed using MetaboScape^®^ 4.0 software (Bruker Daltonics Inc., Bremen, Germany), and Global Natural Products Social Molecular Networking (GNPS, http://gnps.ucsd.edu, accessed in 15 September 2023) was used for tandem mass spectrometry (MS/MS) data identification [36].

Metabolomic data interpretation of the relative abundance of each ion was performed using Principal Component Analysis (PCA) with OriginPro 2025 software, and heatmap visualization was carried out using GraphPad Prism 8.0.2 software (GraphPad Software, Inc., San Diego, CA, USA).

#### 2.5.2. Total Phenolic Content (TPC)

The total phenolic content in the crude extracts (10 mg/mL) was quantified following the method described by Landeta-Salgado et al., 2024 [33]. Results are expressed as milligrams of gallic acid equivalents per 100 g of dry weight (mg GAE/g DW).

#### 2.5.3. pH Variation of Crude Extracts

Crude extracts of *A. cruciatus* grown on glucose were reconstituted with 0.3% (*v*/*v*) DMSO to a concentration of 10 mg/mL. The pH of the extracts was varied by adding 1 M of KOH or HCl solution.

#### 2.5.4. Quantification of Emodin Compound in Crude Extracts

The emodin concentration in the AG extract was determined through HPLC with a photodiode array detector (DAD) (Shimadzu, Kyoto, Japan) using an Onyx Monolithic C18 reverse phase column (5 µm × 100 mm, 4.6 mm, Phenomenex, Torrance, CA, USA), following the method of Qiu et al., 2021 [37] with slight modifications. Emodin standard (MEDCHEM, Fermelo Biotec, Santiago, Chile) concentrations were prepared in solutions ranging from 0.02 to 0.005 mg/mL, and detection was performed at 310 nm. The gradient elution consisted of water and methanol as mobile phases: 0–13 min, 10–100% CH₃OH, 13.01–18 min, 100% CH₃OH, 18.01–18.5 min, 100–10% CH₃OH, and 18.51–35 min, 10% CH₃OH. The flow rate was 0.32 mL/min, with a column temperature of 25 °C and an autosampler temperature of 20 °C. Emodin concentration was expressed as mg/g dry weight (DW) of biomass.

#### 2.5.5. Bioactivity Assay

##### Antioxidant Activity In Vitro

The antioxidant capacity of AG, AM, AD, and seaweed extracts (1 mg/mL) was measured using the Total Antioxidant Capacity Assay Kit (MAK187, Sigma-Aldrich, Burlington, MA, USA). Results are expressed as micromoles of Trolox equivalent antioxidant capacity per gram of sample (µmol TEq/g).

##### Determination of the Cytotoxic Effect on the Neuronal Cell Line

The cytotoxicity of *A. cruciatus* crude extracts on the PC12 cell line was determined through 3-[4,5-dimethylthiazol-2-yl]-2,5 diphenyl tetrazolium bromide (MTT) assay, as described by Gallardo-Fernández et al., 2023 [38]. A 96-well plate was seeded with 3 × 10^4^ cells per well and incubated for 24 h at 37 °C in 5%CO_2_. AG, AD, AM, M, and D crude extracts were dissolved in 100% DMSO to reach a concentration of 50 mg/mL. Crude extracts (AG, AD, AM, M, and D) were dissolved in 100% DMSO to achieve a concentration of 50 mg/mL and then diluted with culture medium to final concentrations of 10, 25, 50, and 100 μg/mL. DMEM with 0.1% DMSO served as a negative control. The cells were incubated with the extracts for 24 h at 37 °C with 5% CO_2_. Finally, cell viability using the MTT assay was measured at 595 nm using a microplate reader (Synergy HT, Biotek, Winooski, VT, USA), based on the protocol described by Gallardo-Fernández et al., 2023 [38].

### 2.6. Statistical Analysis

Statistical analyses were conducted using GraphPad Prism 8.0.2 software (GraphPad Software, Inc., San Diego, CA, USA). All experiments were performed in triplicate, and data are expressed as the mean ± standard deviation (SD). Statistical significance was determined using Tukey’s one-way ANOVA, with a threshold of *p* < 0.05.

## 3. Results

### 3.1. Kinetic Growth of A. cruciatus on Different Culture Media

**Figure 2:**
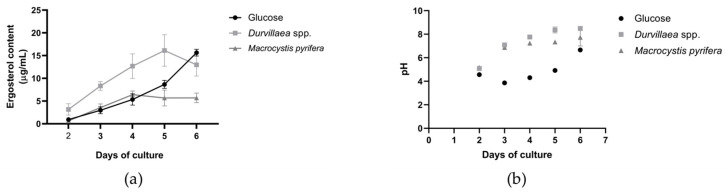
Growth of *A. cruciatus* on different culture media. (**a**) Ergosterol concentration’s (**b**) pH variation after 2, 3, 4, 5, and 6 days of fungal culture on glucose (20 g/L), *Durvillaea* spp. (30 g/L), or *M. pyrifera* (30 g/L) as carbon sources (*n* = 3).

The growth of *A. cruciatus* on glucose and seaweed-based culture media was assessed by measuring the ergosterol content in the fungal biomass (μg/mL) and the variation in pH of the culture medium (Figure 2). The ergosterol concentration was determined through HPLC-DAD after 2, 3, 4, 5, and 6 days of cultivation on AG, AD, and AM media. The growth of *A. cruciatus* on glucose and *Macrocystis pyrifera* media was similar until the fourth day, after which growth in *Durvillaea* spp. medium was higher (Figure 2a). Between days 4 and 6, fungal growth in all seaweed media stabilized. At the end of the culture period, ergosterol concentrations were 15.64 μg/mL for glucose, 12.97 μg/mL for *Durvillaea* spp., and 5.7 μg/mL for *M. pyrifera*.

In terms of pH variation (Figure 2b), the pH of the glucose medium decreased by day 3, reaching a final value of 6.6 at the end of the culture. In contrast, the pH of the seaweed media increased, reaching final values of 8.5 for *Durvillaea* spp. and 6.7 for *M. pyrifera*.

### 3.2. Effects of Different Carbon Sources on the Fungal Metabolome

**Figure 3:**
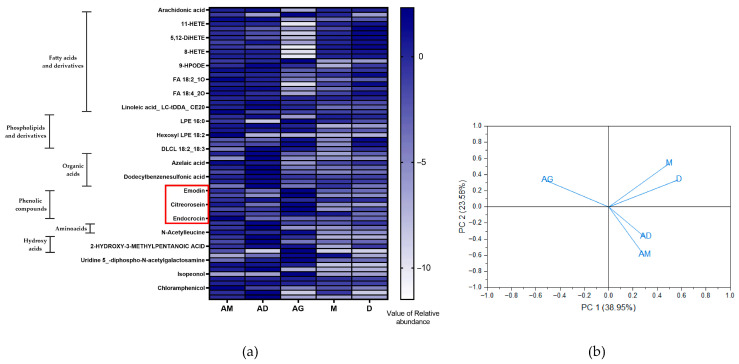
Metabolomic data of *A. cruciatus* extracts grown with different carbon sources based on HPLC-MS/MS analysis. (**a**) Heatmap of metabolites in the crude extracts. Rows represent metabolites, and columns represent different extracts. White and blue boxes represent higher and lower metabolite abundance, respectively. The red box highlights anthraquinone pigments (citreorosein, emodin, and endocrocin). (**b**) Principal Component Analysis (PCA) of metabolite profiles of crude extracts. Score plots show the differential metabolites along Principal Components 1 and 2 (*n* = 2).

The metabolite profiles of AG, AD, and AM crude extracts were analyzed using ESI (−/+) HRMS/MS. A total of 136 metabolites were identified through negative and positive ionization (Figure S1); a summary of the metabolites is presented in Figure 3a. These metabolites were identified based on the similarity of their isotopic patterns and their fragmentation profiles. Heatmap analysis (Figure 3a) showed differences in metabolite regulation between the AG, AD, and AM extracts, with distinct upregulation or downregulation observed in various metabolites. The effect is represented by blue and white boxes in the heatmap, with blue indicating the presence of each metabolite in the sample. Positive values (0 to 2.12) correspond to the detected levels of each metabolite.

A one-to-one analysis of the metabolites identified lipids, such as fatty acids, phospholipids, phenolic compounds, organic acids, amino acids, and hydroxy acids. This classification is represented in Figure 3a. The clustering of fatty acid compounds showed similar relative abundance across the AM, AD, M, and D extracts. However, the abundance of fatty acids and organic acid compounds in the AG extract differed from that in the AM and AD extracts. Clustering of phenolic compounds, amino acids, and hydroxy acids showed higher abundances in the AM and AD extracts compared to their corresponding seaweed medium. Positive values of relative abundance indicated the presence of emodin and citreorosein in the AG extract and citreorosein and endocrocin in the AM extract. The compounds emodin, citreorosein, and endocrocin were not detected in the AD, M, or D extracts (Figure 3a). Seaweed metabolites did not show significant differences; however, some phospholipids and fatty acids were more abundant in one seaweed species than the other.

Principal Component Analysis (PCA) of the metabolomic data revealed that the metabolomes of *A. cruciatus* grown on both seaweed media were clustered together and differed from the metabolome of glucose-grown cultures (Figure 3b). The separation along PC1 (41.04%) indicated distinct differences between the seaweed and glucose media, while separation along PC2 (21.91%) further distinguished the metabolic profiles of the seaweed cultures (AD and AM) from the glucose-grown culture (AG).

### 3.3. Characterization of the Crude Extracts from A. cruciatus

The characterization of crude extracts from *A. cruciatus* grown on glucose, *Durvillaea* spp., and *M. pyrifera* was performed by estimating the total phenolic content (TPC), the total antioxidant capacity (TAC), and cytotoxicity.

#### 3.3.1. Total Phenolic Content and In Vitro Antioxidant Capacity

The highest TPC and TAC values were observed in the AG extract, which had 3.09 ± 0.04 mg GAE/g DW and 39.70 ± 1.0 µmol TEq/g biomass, respectively (Table 1).

The emodin content in the crude extract was quantified through HPLC-DAD using a pure emodin standard. Emodin concentration was determined only in the AG extract, as this compound was not detected in the AD, AM, M, or D extracts (Figure S2). The concentration of emodin in the AG extract was 0.36 ± 0.08 mg/g dry weight (DW) biomass.

**Figure 4:**
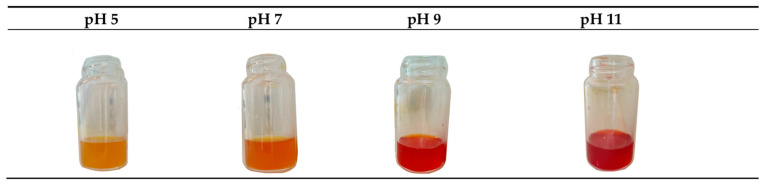
Characterization of crude extracts from *A. cruciatus* grown on glucose medium. Color change of the extract at different pH levels.

The crude extract of *A. cruciatus* grown on a glucose medium exhibited a yellow color (Figure 4), which changed to orange and red as the pH increased. When the pH decreased with the HCl, the yellow color was restored. This effect was not observed in the AD and AM extracts due to the brown color of the extracts.

#### 3.3.2. Cytotoxicity of Crude Extracts on Neuronal Cell Lines

**Figure 5:**
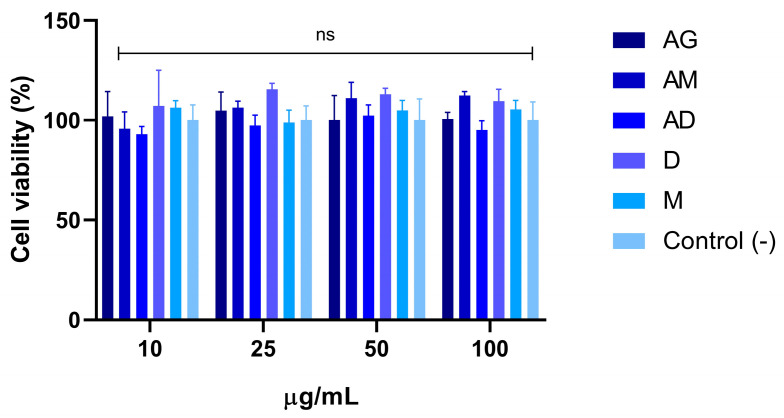
Cytotoxicity evaluation of crude extracts on neuronal cell lines. Cell viability percentages of PC12 cells relative to untreated control cells (control -). Results are expressed as the mean ± standard deviation. DMSO 0.1% served as the negative control. ns: not significant (*n* = 2).

The cytotoxicity of crude extracts was evaluated using the MTT assay on PC12 neuronal cells. Cell viability percentages are presented relative to untreated control cells (control-). The results showed no significant reduction in cell viability at any of the tested concentrations of AG, AD, or AM extracts (Figure 5). The viability of PC12 cells was comparable to the negative control (DMSO 0.1%).

## 4. Discussion

### 4.1. Variation in the Growth of A. cruciatus with Different Carbon Sources

The use of brown seaweed in biorefineries is gaining attention due to its high content of polysaccharides, including laminarian, alginate, cellulose, and fucoidan, which are integral components of the cell wall [19]. The concentration and polymerization of these polysaccharides, as well as their monomers, vary between species [17]. For example, the alginate monomers guluronic acid (G) and mannuronic acid (M) can polymerize into different forms, including poly-M, poly-G, or a mixed M/G structure [39].

Ergosterol content was measured in the fungal biomass due to its specificity as a growth parameter for fungi [12,13]. This methodology was ideal for assessing the growth of the filamentous fungus *Asteromyces cruciatus*, which exhibits growth in a liquid culture medium in the form of unamorphous filamentous spheres. Additionally, it allows for comparisons of growth between different cultures, as estimating fungal growth solely by dry weight biomass could be influenced by variations in the culture medium, potentially affecting the final weight. The growth of *A. cruciatus* on the different media was ended on day 6, as at this point the fungus grown on glucose-based medium had consumed all available glucose (Figure S1). The comparison of metabolite diversity was then evaluated at this same time point. The growth of *A. cruciatus* on culture media derived from *Durvillaea* spp. and *Macrocystis pyrifera* suggests that this fungus is capable of assimilating and degrading the polysaccharides present in brown seaweed. A previous study demonstrated that the fungi can grow on an alginate medium [21], indicating that the fungal growth observed on both seaweed species may be attributed to the alginate content in their cell walls [11,12]. The variation in *A. cruciatus* growth on the two seaweed cultures may be attributed to differences in polysaccharide concentrations within the seaweed, as well as variations in enzymatic degradation processes. These processes are influenced by the specific activity of fungal polysaccharide lyases and their substrate specificity, which includes the polymerization patterns of the polysaccharides [40]. To better understand how this fungus degrades seaweed and assimilates its polysaccharides, it is necessary to identify the production of enzymes, such as alginate lyases, cellulases, and fucoidanases [41], or to estimate the carbohydrate composition using high-performance anion-exchange chromatography (HPAEC) [42].

On the other hand, pH variations during glucose metabolism exhibit a bimodal acidification–basification pattern (Figure 2b). This behavior could be attributed to an excess of glucose during the first 0–3 days of culture, when the initial growth conditions start with 20 g/L of glucose (day 0), decreasing to 15 g/L by day 3 (Figure S2). This excess glucose induces metabolism via the oxidation of glucose to gluconic acid, which lowers the pH of the medium. From days 4 to 6, the glucose concentration decreases from 10 g/L to 0 g/L, which may shift metabolism toward alkaline conditions due to carbon source deprivation, catalyzed by the deamination of non-preferred carbon sources. These bimodal pH fluctuations have been previously described in a pathogenic fungus [43].

In contrast, the pH of seaweed cultures increases throughout the growth period. This behavior could be related to the assimilation of mannuronic and guluronic acids from the degradation of alginate polysaccharides. Specifically, the acids present in the medium may act as neutralizing compounds, and their consumption could increase the pH. This result is consistent with the findings of Schaumann and Weide 1990 [21], who reported that alginate assimilation increases the pH of the culture medium.

### 4.2. The Effect of Different Carbon Sources on the Functional Properties of the Fungal Metabolome

The metabolome of *A. cruciatus* was influenced by the type of carbon source used in the medium (Figure 3). The chemical composition of the media derived from seaweed and glucose differs, which affects the fungal metabolome profile. A comparative analysis of the metabolite data led to the identification of the anthraquinone compounds emodin, citreorosein, and endocrocin in *A. cruciatus* extracts (Figure 3a). These compounds are natural orange–yellow pigments found in plants, molds, and lichens [44,45]. Some research on bioactive components indicates that citreorosein modulates inflammatory diseases [46,47], and emodin has demonstrated anticancer, anti-inflammatory, antioxidant, and antimicrobial bioactivity [37]. Through an untargeted metabolomic approach, approximately 43% of the total metabolites were identified as lipids or lipid-derived compounds, while 11% were identified as phenolic compounds, such as emodin, endocrocin, citreorosein, aurapte, and vanillin-4-sulfate, among others. For more specific identification of the phenolic compounds, it may be beneficial to modify the mass spectrometry methodology, such as the mobile phase or the gradient conditions.

The relative abundance of metabolites, indicated by the blue and white cells in Figure 3a, shows that citreorosein and emodin are more abundant when *A. cruciatus* grows on a glucose medium. On the other hand, endocrocin is more abundant when the fungus grows on *Macrocystis pyrifera* medium. These results suggest that the culture medium influences pigment synthesis, with variations even between the two brown seaweed species. The synthesis of these pigments is more closely related to fungal metabolism rather than the algae itself. The highest phenolic content and antioxidant capacity were observed in the AG extracts, which is consistent with the heatmap results, where phenolic compounds are more abundant than in the AM and AD extracts. Furthermore, the influence of the culture medium affects the lipid content in crude extracts, with AM and AD exhibiting higher phospholipid and fatty acid content than AG and slightly more than their respective seaweed sources. This effect may be attributed to the metabolism of the seaweed and its assimilation into the fungal biomass.

Cluster analysis of the metabolomic data (Figure 3b) reveals that the fungal metabolite profiles of *A. cruciatus* grown on both seaweed species are relatively similar, in contrast to the profile of the fungus grown on glucose. This similarity was also observed in the total phenolic content and the antioxidant capacity, where *A. cruciatus* grown on both seaweeds produced similar results distinct from those observed in the glucose medium. This notable difference between seaweed and glucose metabolism may be due to the complexity of macro- and micronutrients present in the seaweed, such polysaccharides, fatty acids, proteins, vitamins, and minerals [17,48,49]. To further understand how this fungus metabolizes these substrates and its overall behavior, a transcriptomic approach and genome sequencing are necessary.

On the other hand, no significant differences in toxicity were observed in the neuronal cell line assay, suggesting that the extracts did not contain toxic compounds or that the concentration was too low to exhibit any toxicity. Similar studies of fungal crude extracts have been evaluated on other cell lines, with varying effects in each study. Some studies showed detrimental effects on specific cell lines, while others showed no toxic effects. The results depend on various factors, such as the type of cell line, the concentration, and the characteristics of the crude extract [50,51]. For future bioactivity assays, it would be of interest to evaluate the effects of these crude extracts on carcinogenic and epithelial cell lines, particularly considering that some anthraquinone compounds exhibit diverse bioactivity effects.

The characterization of the fungal extracts based on their functional properties, such as their total phenolic content, in vitro antioxidant capacity, and cytotoxicity assays, allows us to explore how these extracts could be utilized in a biotechnology context. Currently, there is increasing interest in the production and identification of metabolites, such as alkaloids, anthraquinone [1] fatty acids, docosahexaenoic acid, eicosapentaenoic acid, and linoleic acid [52], which have significant antioxidant properties and no detrimental effects.

### 4.3. Basic pH Solution Changes the Color of the Extract

The demand for natural pigments with stable and interesting properties in industrial applications is growing. The crude extract of *A. cruciatus* grown on a glucose medium was sensitive to pH variations, changing from yellow to intense red as the pH increased (Figure 4). This result suggests that the pH-dependent behavior is due to the radical groups in the chemical structure of the anthraquinone compounds, which are capable of reduction or oxidation depending on the pH. This color shift is consistent with other investigations of anthraquinone compounds [53,54]. These properties suggest that *A. cruciatus* extracts could potentially be used as pH indicators.

## 5. Conclusions

This study demonstrates that *Asteromyces cruciatus* is capable of degrading and utilizing polysaccharides from brown seaweeds. The fungus exhibited different growth patterns and pH modulation when cultured on seaweed-derived media compared to glucose, suggesting adaptive metabolic responses to varying carbon sources. The functional properties of crude extracts grown in glucose medium are superior to seaweed medium in terms of phenolic content and antioxidant properties. Metabolomic analysis revealed that *A. cruciatus* synthesizes distinct anthraquinone compounds, such as emodin, citreorosein, and endocrocin, with production levels varying depending on the carbon source. These findings highlight the potential of *A. cruciatus* for biotechnological applications, including the production of bioactive compounds, natural pigments, and the use of unconventional raw materials. Additionally, the pH-dependent color changes observed suggest the potential use of these compounds as pH indicators. Future research could focus on the purification of these compounds, their analysis in other cell lines, and their scalable production in seaweed-based culture media.

## Figures and Tables

**Table 1:** Total phenolic content (TPC) and antioxidant activity (TAC) of *A. cruciatus* grown on glucose, *Durvillaea* spp., and *Macrocystis pyrifera*.

Sample	TPC (mg GAE ^1^/g DW)	TAC (µmol TEq ^1^/g biomass)
AG	3.09 ± 0.04 ^a^	39.70 ± 1.0 ^a^
AM	1.19 ± 0.11 ^b^	23.68 ± 3.2 ^b^
AD	1.21 ± 0.17 ^b^	16.66 ± 1.5 ^b^
M	1.15 ± 0.13 ^b^	14.49 ± 3.6 ^b^
D	0.83 ± 0.04 ^b^	19.35 ± 2.4 ^b^

^1^ GAE: Gallic acid equivalent; TEq: Trolox equivalent. ^a, b^ letters indicate significant differences (*p* < 0.05) in the parameter evaluated.

## Data Availability

The original contributions presented in the study are included in the article; further inquiries can be directed to the corresponding author.

## Supplementary Materials

The following supporting information can be downloaded at https://www.mdpi.com/article/10.3390/jof11010003/s1. Figure S1: HPLC-DAD chromatograms of the crude extracts from *A. cruciatus* grown on glucose (AcG), *Durvillaea* spp. (AcD), and *M. pyrifera* (AcM), as well as their respective seaweed media (Med M and Med D). Emodin (STD Emo) at 0.005 mg/mL was used as a standard for comparison. Measurements were performed at 310 nm using a photodiode array detector; Figure S2: Ergosterol and glucose concentrations of *A. cruciatus* grown on glucose medium for 2, 3, 4, 5, and 6 days. Initial growth conditions: 20 g/L glucose (*n* = 3).; Table S1: Metabolomic data from positive (ESI+) and negative ionization (ESI−).
