# Supplementary material for: Assessment of the Chemical Diversity and Functional Properties of Secondary Metabolites from the Marine Fungus Asteromyces cruciatus
Source: J Fungi (Basel). 2024 Dec 24;11(1):3. doi: 10.3390/jof11010003 (PMC11766682; doi:10.3390/jof11010003)
Supplement: Supplementary file 1 [file jof-11-00003-s001.zip › jof-3344561-supplementary.pdf]

Journal of *Fungi*

Article

Assessment of the Chemical Diversity and Functional Properties of Secondary Metabolites from the Marine Fungus *Asteromyces cruciatus*

María Paz González-Troncoso [1], Catalina Landeta-Salgado [1], Javiera Munizaga [1], Ruth Hornedo-Ortega [2], María del Carmen García-Parrilla [2] and María Elena Lienqueo [1,*]

[1] Department of Chemical Engineering, Biotechnology, and Materials, Centre for Biotechnology and Bioengineering (CeBiB), University of Chile, Beauchef 851, Santiago 8370456, Chile; maria.gonzalez.t@ug.uchile.cl (M.P.G.-T.); cmlandeta@uc.cl (C.L.-S.); jmmunizaga@uc.cl (J.M.)
[2] Departamento de Nutrición y Bromatología, Toxicología y Medicina Legal, Facultad de Farmacia, Universidad de Sevilla, C/Profesor García González nº 2, 41012 Sevilla, Spain; rhornedo@us.es (R.H.-O.); mcparrilla@us.es (M.d.C.G.-P.)
* Correspondence: mlienque@uchile.cl

Supplementary Materials:

Table S1. Metabolomic data from positive (ESI +) and negative ionization (ESI -).

ESI +									
**Bucket label**	**RT**	**m/z**	**Name**	**Formula**	**AM**	**AD**	**AG**	**M**	**D**
495,33731 Da 762,62 s	12,71	496,34539	_2-_3-_hexadecanoyloxy_-2-hydroxypropyl phosphonato_oxy_ethyl_trimethylazanium	C24H50NO7P	8041,5	4318,5	15703,5	11384	518
509,35131 Da 834,99 s	13,92	510,35859	1-heptadecanoyl-2-hydroxy-sn-glycero-3-phosphocholine	C25H52NO7P	2084	580	1241	459,5	0
521,35072 Da 806,87 s	13,45	522,358	1-Oleoyl-sn-glycero-3-phosphocholine	C26H52NO7P	119121,5	103817	267362	52327,5	2765
453,28819 Da 769,84 s	12,83	454,29547	1-palmitoyl-2-hydroxy-sn-glycero-3-phosphoethanolamine	C21H44NO7P	6774	409,5	65956	15448	695,5
481,31794 Da 732,53 s	12,21	482,32522	1-pentadecanoyl-2-hydroxy-sn-glycero-3-phosphocholine	C23H48NO7P	0	0	0	6093,5	0
523,36516 Da 887,67 s	14,79	524,37243	1-Stearoyl-sn-glycero-3-phosphocholine	C26H54NO7P	7132,5	3315	29663,5	3015,5	502
301,29677 Da 720,75 s	12,01	302,30404	2,2_-_Tetradecylimino_diethanol	C18H39NO2	0	0	0	0	0
145,05312 Da 230,83 s	3,85	146,0604	3-Formylindole	C9H7NO	648,5	0	0	0	336
145,08482 Da 59,12 s	0,99	146,09209	4-GUANIDINOBUTANOATE	C5H11N3O2	0	0	0	0	484,5
203,11596 Da 67,52 s	1,13	204,12323	Acetylcarnitine	C9H17NO4	0	22961	5145,5	0	538
267,09733 Da 60,47 s	1,01	268,10461	Adenosine	C10H13N5O4	4084	3496,5	14153	370,5	5190
290,22151 Da 1052,62 s	17,54	291,22879	Androsterone	C19H30O2	17376,5	23601,5	330,5	985	1700
174,11136 Da 45,36 s	0,76	175,11864	Arginine	C6H14N4O2	6066,5	3841,5	0	0	0
390,27756 Da 1172,58 s	19,54	391,28484	BIS_2-ETHYLHEXYL_PHTHALATE	C24H38O4	78071	230529	101706	110855	0
161,10435 Da 62,54 s	1,04	162,11163	Carnitine _Dl_	C7H15NO3	35700	0	5108	4416,5	3961,5
342,28821 Da 576,45 s	9,61	343,29549	CocamidopropylBetaine	C19H38N2O3	9932,5	10325	9911	6124	4676,5
145,11003 Da 55,54 s	0,93	146,11731	DEOXYCARNITINE	C7H15NO2	11664	4966	1069,5	16368	76006
512,30078 Da 630,24 s	10,5	495,29742	Dereplicator Identification - Monogalactosylmonoacylglycerol_Hemolysi	C27H44O9	48502,5	16694,5	0	4167	5309,5
197,11928 Da 69,72 s	1,16	198,12656	Dibenzylamine	C14H15N	31352	28834,5	5791,5	29653,5	0
222,08951 Da 587,66 s	9,79	245,07839	Diethyl-phthalate	C12H14O4	2829,5	9734	11997,5	7527,5	8418
278,15140 Da 851,80 s	14,2	279,15867	Di-n-butyl phthalate	C16H22O4	25809	28978	0	21628	15524
302,22465 Da 952,70 s	15,88	303,23193	Eicosanoids_EPA_C20H30O2	C20H30O2	34814	27537,5	0	36184	34053
282,25552 Da 885,38 s	14,76	283,26255	Elaidic acid	C18H34O2	1018,5	1214,5	0	0	0
229,08842 Da 717,82 s	11,96	230,0957	Ergothioneine	C9H15N3O2S	0	0	2086,5	0	0
337,33436 Da 1192,62 s	19,88	338,34163	Erucamide	C22H43NO	104037	178675,5	246196,5	192955	69403
278,22535 Da 791,03 s	13,18	279,23135	gamma-Linolenic acid	C18H30O2	154032	72624,5	10113,5	14185,5	22279,5
146,06509 Da 45,91 s	0,77	147,07236	Glutamine	C5H10N2O3	3440	7966,5	0	0	725
465,31111 Da 502,69 s	8,38	466,31838	Glycocholic acid	C26H43NO6	13052	0	0	0	0
449,31290 Da 606,60 s	10,11	450,32018	Glycoursodeoxycholic acid	C26H43NO5	6448,5	0	0	0	0
302,22496 Da 837,13 s	13,95	303,23224	h_128_methyl_1_testosterone	C20H30O2	0	2476,5	0	50156	44772
276,20808 Da 714,92 s	11,92	277,21536	h_14_19_norandrosterone	C18H28O2	2991	1882,5	1880,5	478,5	848
316,24091 Da 954,35 s	15,91	317,24818	h_268_isomere_de_oxymetholone_m2	C21H32O2	15667	27613,5	0	7235	18533,5
304,23964 Da 1008,30 s	16,81	305,24691	h_31_mesterolone	C20H32O2	37672	26221,5	0	34181	31520
288,20933 Da 841,50 s	14,03	289,21661	h_51_boldenone-m	C19H28O2	11295	26224	0	871	0
155,06841 Da 44,87 s	0,75	156,07568	Histidine	C6H9N3O2	0	18055,5	5362,5	0	0
294,21721 Da 535,24 s	8,92	295,22449	Hydroxy octadecatrienoic acid	C18H30O3	795	930	0	0	0
159,06886 Da 65,31 s	1,09	160,07614	Indole-3-acetaldehyde	C10H9NO	1962	0	775	0	0
231,14542 Da 69,59 s	1,16	232,1527	Isobutyryl-carnitine_ LC-tDDA_ CE20	C11H21NO4	0	9531	0	0	0
131,09434 Da 61,06 s	1,02	132,10161	Isoleucine	C6H13NO2	10536,5	11060	12973,5	2948,5	24021,5
273,26660 Da 647,01 s	10,78	274,27388	Lauryldiethanolamine	C16H35NO2	0	0	0	636	454
161,10445 Da 50,20 s	0,84	162,11173	L-Carnitine	C7H15NO3	0	56219,5	3844,5	2442,5	5647,5
230,16615 Da 60,54 s	1,01	231,17342	Leucylvaline	C11H22N2O3	1924,5	3012	4393	0	0
280,23901 Da 826,58 s	13,78	281,24526	Linoleic acid	C18H32O2	1895	2108	0	1053,5	1571,5
523,36765 Da 868,75 s	14,48	524,37492	Lyso-PC 18:0	C26H54NO7P	806	0	2806,5	0	782
521,34593 Da 769,25 s	12,82	522,3532	Lyso-PC 18:1_9Z_	C26H52NO7P	0	6485	2500,5	0	1501,5
356,29217 Da 766,28 s	12,77	357,29945	monoolein	C21H40O4	3225,5	12924	0	0	0
271,25096 Da 818,95 s	13,65	272,25824	Myristoyl Ethanolamide	C16H33NO2	0	59747,5	0	1450	0
244,17588 Da 64,65 s	1,08	245,18316	N-Leucyl-leucine_ AIF_ CE10_ MS2Dec	C12H24N2O3	2253	1705	7298	677,5	0
799,50242 Da 990,31 s	16,51	800,5097	PC 16:4_22:5	C46H74NO8P	0	0	0	0	0
829,54403 Da 866,58 s	14,44	830,5513	PC 20:4_20:4	C48H80NO8P	1454	1331	0	0	0
537,34803 Da 623,00 s	10,38	538,35531	PC 9:0_9:0	C26H52NO8P	3063,5	1950,5	0	0	1241,5
467,30326 Da 667,05 s	11,12	468,31054	PC O-14:0	C22H46NO7P	23881	0	0	20812,5	0
493,31675 Da 696,01 s	11,6	494,32403	PC O-16:1	C24H48NO7P	31950,5	2851,5	7262,5	1640,5	0
507,33211 Da 742,90 s	12,38	508,33939	PC O-17:1	C25H50NO7P	8298	0	0	0	0
541,31727 Da 687,96 s	11,47	542,32455	PC O-20:5	C28H48NO7P	210029	1407	0	34858	0
569,33862 Da 345,34 s	5,76	570,34589	PC O-22:5	C30H52NO7P	3324,5	386,5	0	0	0
495,33499 Da 781,30 s	13,02	496,34227	PC_16:0_0:0_	C24H50NO7P	46920	9703,5	204684	96914	1914
759,56317 Da 932,17 s	15,54	760,57045	PC_16:0_18:1_9Z_	C42H82NO8P	4545	3061,5	0	7081,5	0
278,16492 Da 64,89 s	1,08	279,1722	Phe-Ile	C15H22N2O3	0	1325,5	2789,5	842	0
592,26990 Da 1049,17 s	17,49	593,27718	Pheophorbide A	C35H36N4O5	15617	338838	0	22790,5	356450
592,26821 Da 1070,61 s	17,84	593,27549	Pheophorbide A	C35H36N4O5	0	80037,5	0	0	83995,5
148,01611 Da 858,10 s	14,3	149,02339	Phthalic anhydride	C8H4O3	60538	78853,5	0	41957,5	45332
317,29271 Da 742,69 s	12,38	318,29998	Phytosphingosine	C18H39NO3	11041,5	0	1055,5	487,5	0
276,13233 Da 45,66 s	0,76	277,13961	saccharopine	C11H20N2O6	433,5	3408	3922	0	0
145,15735 Da 46,16 s	0,77	146,16463	Spermidine	C7H19N3	3335,5	0	64960,5	0	839,5
182,07126 Da 293,46 s	4,89	183,07854	Triethyl phosphate	C6H15O4P	33007,5	22131	24750	22355	20392
266,16501 Da 779,50 s	12,99	267,17229	Tri-isobutylphosphate	C12H27O4P	835,5	0	0	0	0
245,16315 Da 60,01 s	1	246,17043	Valeryl-carnitine_ AIF_ CE30_ MS2Dec	C12H23NO4	13152,5	4728,5	9881	1082,5	14478,5

ESI (-)									
Bucket label	RT	m/z	Name	Formula	AM	AD	AG	M	D
481,31839 Da 862,23 s	14,37	480,31111	_2-aminoethoxy_2-hydroxy-3-_octadecanoyloxy_propoxy_phosphinic acid	C23H48NO7P	1090	0	7735	2240	0
328,17213 Da 473,40 s	7,89	327,16485	_3E,12E_-3,12-dimethyl-8-methylidene-6,18-dioxatricyclo_14.2.1.0_,_nonadeca-3,12,16_19_-triene-7,17-dione	C20H24O4	1482,5	3190,5	1529	0	0
300,26713 Da 1053,67 s	17,56	299,25986	_R_-2-hydroxystearic acid	C18H36O3	1370	9580	235,5	337,5	420
330,23976 Da 478,32 s	7,97	329,23333	_Z_-5,8,11-trihydroxyoctadec-9-enoic acid	C18H34O5	69037	33116,5	598	2335	5869,5
320,23480 Da 810,44 s	13,51	319,22752	11-HETE	C20H32O3	42244,5	17258	0	36536	62946,5
300,26608 Da 882,19 s	14,7	299,25908	12-Hydroxyoctadecanoic acid	C18H36O3	1399,5	1735	253	771,5	611
294,21832 Da 736,39 s	12,27	293,21104	13-HOTrE	C18H30O3	14487	1840	477,5	7631	97034
318,21908 Da 736,14 s	12,27	317,2118	18-HEPE	C20H30O3	10005	1561,5	0	10801	36429
382,21183 Da 705,11 s	11,75	381,20455	1-Myristoyl-2-hydroxy-sn-glycero-3-phosphate	C17H35O7P	1586,5	1886	0	2404,5	1871
338,24578 Da 795,37 s	13,26	337,2385	2-_2-hydroxybut-3-en-2-yl_-3a,6,6,9a-tetramethyl-2,4,5,5a,7,8,9,9b-octahydro-1H-benzo_e_1_benzofuran-4,5-diol	C20H34O4	0	3124,5	1185	0	328,5
523,29151 Da 760,12 s	12,67	522,28424	2-amino-3-_hydroxy-_2-hydroxy-3-_octadec-9-enoyl_oxypropoxy_phosphoryl_oxypropanoic acid	C24H46NO9P	0	8810,5	1703,5	0	0
218,05540 Da 59,02 s	0,98	217,04812	2-Benzyl-4-chlorophenol	C13H11ClO	42825	54054,5	0	82093	0
132,07903 Da 69,10 s	1,15	131,07175	2-HYDROXY-3-METHYLPENTANOIC ACID	C6H12O3	2144	3412	6013	0	1691,5
246,02050 Da 64,10 s	1,07	245,01322	3-_3-Hydroxyphenyl_propionic acid sulfate	C9H10O6S	3312	0	66486,5	0	0
146,06888 Da 61,40 s	1,02	145,06161	3-Ureidoisobutyric acid_ LC-tDDA_ CE10	C5H10N2O3	2700,5	4743,5	509	0	639
336,22927 Da 662,07 s	11,03	335,22199	5,12-DiHETE	C20H32O4	5510,5	593,5	0	7212,5	19037,5
336,22988 Da 731,82 s	12,2	335,2226	5,6-DiHETE	C20H32O4	1060,5	1926,5	0	2455	5028,5
318,21886 Da 774,45 s	12,91	317,21167	5-HEPE	C20H30O3	20160	1986,5	0	35920	77529,5
320,23531 Da 831,56 s	13,86	319,22694	5-HETE	C20H32O3	26077,5	7387,5	0	74947	151581
139,02709 Da 70,55 s	1,18	138,01982	6-Hydroxynicotinic acid	C6H5NO3	0	1025	0	299	0
320,23474 Da 783,02 s	13,05	319,22746	8-HETE	C20H32O3	3007,5	2654,5	0	3431,5	8335,5
320,23462 Da 827,02 s	13,78	319,22734	9-HETE	C20H32O3	44827,5	19606	0	39624,5	61413
294,21891 Da 866,24 s	14,44	293,21163	9-HOTrE	C18H30O3	1073,5	911	7299,5	0	0
312,23002 Da 803,62 s	13,39	311,22274	9-HPODE	C18H32O4	585,5	477,5	0	0	2059,5
296,23515 Da 784,41 s	13,07	295,22788	9-hydroxy-10,12-octadecadienoic acid	C18H32O3	184615	74816	12087,5	16383,5	49104
304,23979 Da 1006,87 s	16,78	303,23276	Arachidonic acid	C20H32O2	300825	234429,5	2351,5	237054	252477,5
298,16017 Da 612,16 s	10,2	297,15289	aurapten	C19H22O3	576	0	5543	0	0
188,10470 Da 233,35 s	3,89	187,09742	Azelaic acid	C9H16O4	3847,5	1235,5	0	452,5	1127,5
340,20727 Da 840,07 s	14	339,19999	Canrenone	C22H28O3	16935,5	16879,5	12718	5030	4252,5
322,01291 Da 273,90 s	4,57	321,00502	Chloramphenicol	C11H12Cl2N2O5	257474	10527	1930	11429,5	2190,5
286,04780 Da 447,61 s	7,46	285,04052	Citreorosein	C15H10O6	18959	488,5	43449	312,5	161
192,02708 Da 108,47 s	1,81	191,0198	Citric acid	C6H8O7	2220,5	1457,5	169,5	314,5	742
134,02152 Da 57,06 s	0,95	133,01425	D-_-Malic acid	C4H6O5	3670	27974	1918,5	718,5	1619
924,51032 Da 1125,91 s	18,77	923,50304	DLCL 14:1_22:3	C45H82O15P2	166	0	4309,5	0	2196
924,51014 Da 996,24 s	16,6	923,50286	DLCL 16:2_20:2	C45H82O15P2	177	298,5	2933	0	1876
922,49333 Da 975,57 s	16,26	921,48605	DLCL 18:2_18:3	C45H80O15P2	0	0	0	0	927
326,19161 Da 780,77 s	13,01	325,18434	Dodecylbenzenesulfonic acid	C18H30O3S	563,5	169	18990,5	10066,5	0
302,22425 Da 951,03 s	15,85	301,21698	Eicosapentaenoic acid	C20H30O2	190577,5	159651,5	0	138747,5	213519
270,05343 Da 450,64 s	7,51	269,04615	Emodin	C15H10O5	0	0	278800	0	0
314,04269 Da 381,74 s	6,36	313,03542	Endocrocin	C16H10O7	1436,5	0	0	0	0
298,25111 Da 837,88 s	13,96	297,24383	FA 18:1_1O	C18H34O3	18259,5	9685	1693,5	13489	48612,5
296,23465 Da 799,91 s	13,33	295,22737	FA 18:2_1O	C18H32O3	38258	6486	559,5	561,5	1697,5
294,21911 Da 728,55 s	12,14	293,21184	FA 18:3_1O	C18H30O3	25762	11483,5	0	7648,5	55193
310,21446 Da 535,99 s	8,93	309,20718	FA 18:3_2O	C18H30O4	2961,5	635	0	678,5	3262
308,19860 Da 552,69 s	9,21	307,19132	FA 18:4_2O	C18H28O4	5114	1107	0	1005,5	5371,5
342,27820 Da 1141,70 s	19,03	341,27092	FAHFA 18:0_2:0	C20H38O4	0	0	0	0	0
564,50959 Da 1086,26 s	18,1	563,50231	FAHFA 36:1	C36H68O4	14997,5	15505	9561,5	4544	3392,5
182,07908 Da 50,52 s	0,84	181,0718	Galactitol	C6H14O6	9427	21481,5	0	6145,5	0
172,01376 Da 48,53 s	0,81	171,00648	GLYCEROL 2-PHOSPHATE	C3H9O6P	2296,5	5599	0	0	0
639,33799 Da 688,79 s	11,48	638,33071	Hexosyl LPE 18:2	C29H54NO12P	22451	0	0	0	0
326,19137 Da 714,48 s	11,91	325,1841	hydroquinidine	C20H26N2O2	1887	4924	27956	224,5	3795,5
166,06285 Da 277,17 s	4,62	165,05557	Isopeonol	C9H10O3	0	0	12980,5	0	0
266,15503 Da 653,34 s	10,89	265,14775	Lauryl sulfate	C12H26O4S	64542,5	44459,5	42186,5	30706,5	37111
147,05346 Da 59,90 s	1	146,04619	L-Glutamic acid	C5H9NO4	3483	9052,5	1931	974,5	3191
280,23983 Da 1019,31 s	16,99	279,2331	Linoleic acid_ LC-tDDA_ CE20	C18H32O2	505056	625307	420830,5	34642	66077,5
453,28447 Da 761,60 s	12,69	452,27719	LPE 16:0	C21H44NO7P	5589	0	38258	8036	2544,5
477,28583 Da 744,30 s	12,4	476,27855	LPE 18:2	C23H44NO7P	494,5	490,5	546,5	0	1478,5
204,09004 Da 62,41 s	1,04	203,08276	L-Tryptophan	C11H12N2O2	908,5	3163,5	0	0	118,5
278,15133 Da 746,79 s	12,45	277,14406	MEHP	C16H22O4	0	0	361,5	1013,5	0
310,21509 Da 710,06 s	11,83	309,20782	methyl _2E,4E,8E_-7,13-dihydroxy-4,8,12-trimethyltetradeca-2,4,8-trienoate	C18H30O4	5222	8174	0	0	0
173,10531 Da 67,18 s	1,12	172,09803	N-Acetylleucine	C8H15NO3	1849,5	545	3036,5	0	0
353,29379 Da 990,53 s	16,51	352,28651	Oleyl sarcosine	C21H39NO3	247	0	0	0	0
479,30150 Da 772,63 s	12,88	478,29416	Phosphatidylethanolamine lyso 18:1	C23H46NO7P	24237	2741,5	10005,5	0	1916,5
338,21311 Da 825,05 s	13,75	337,20583	Piperonyl butoxide	C19H30O5	5739,5	1940	4726,5	2937,5	1590,5
488,34757 Da 929,05 s	15,48	487,3403	Pygenic acid B b	C30H48O5	0	3296	0	0	0
294,18667 Da 766,65 s	12,78	293,17939	Tetradecylsulfate	C14H30O4S	0	0	14636	12746	0
607,08286 Da 70,49 s	1,17	606,07559	Uridine 5_-diphospho-N-acetylgalactosamine	C17H27N3O17P2	0	198	1155	0	0
607,08264 Da 45,90 s	0,76	606,07536	URIDINE DIPHOSPHATE-N-ACETYLGLUCOSAMINE	C17H27N3O17P2	16204,5	7510,5	11404	0	0
232,00460 Da 61,51 s	1,03	230,99733	Vanillin-4-sulfate	C8H8O6S	4078,5	712,5	10001	7250	353

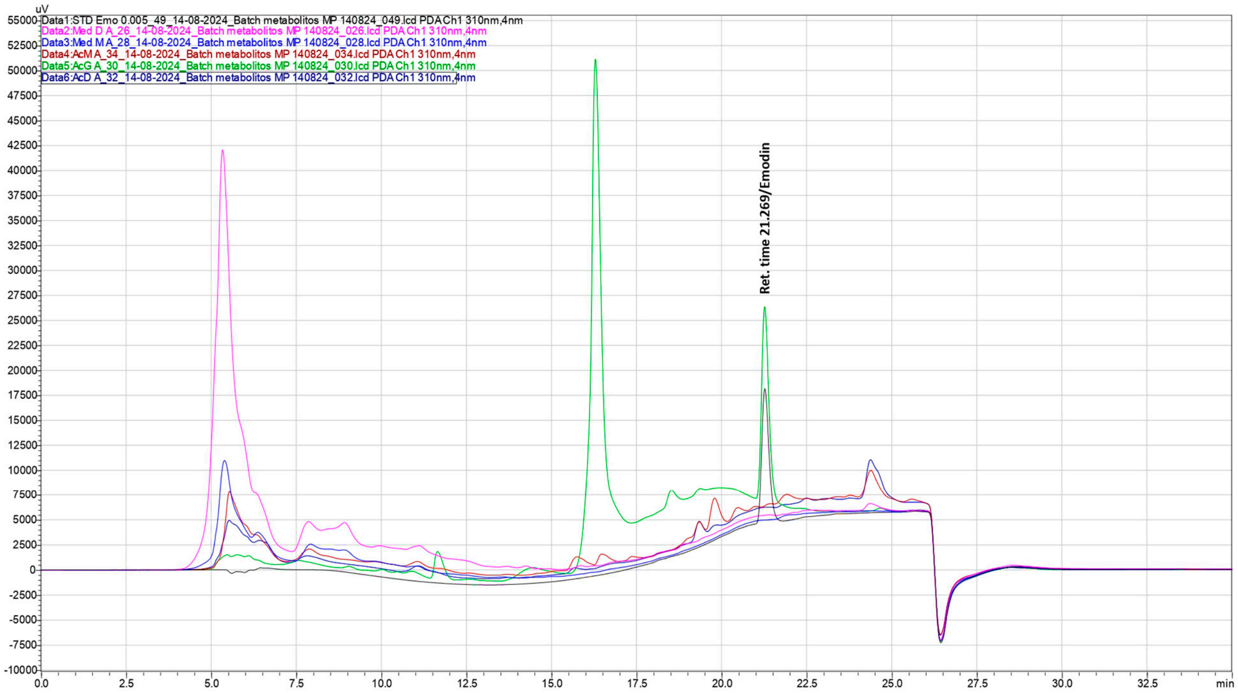

Figure S1: HPLC-DAD chromatograms of the crude extracts from *A. cruciatus* grown on glucose (AcG), *Durvillaea* spp. (AcD), and *M. pyrifera* (AcM), as well as their respective seaweed media (Med M and Med D). Emodin (STD Emo) at 0.005 mg/mL was used as a standard for comparison. Measurements were performed at 310 nm using a photodiode array detector.

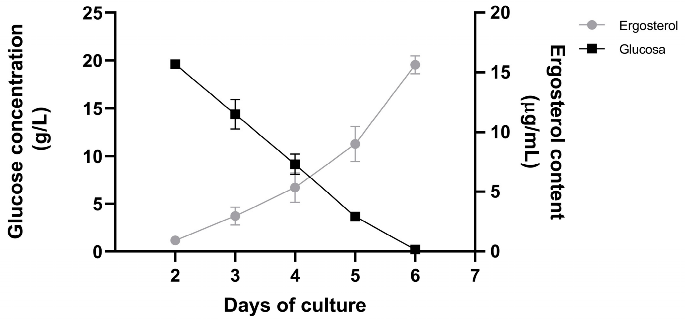

Figure S2: Ergosterol and glucose concentrations of *A. cruciatus* grown on glucose medium for 2, 3, 4, 5, and 6 days. Initial growth conditions: 20 g/L glucose (n = 3).
